# Heterogeneity of metastatic pancreatic adenocarcinoma: Lung metastasis show better prognosis than liver metastasis—a case control study

**DOI:** 10.18632/oncotarget.9861

**Published:** 2016-06-06

**Authors:** Decoster Claire, Gilabert Marine, Autret Aurélie, Turrini Olivier, Oziel-Taieb Sandrine, Poizat Flora, Giovannini Marc, Viens Patrice, Iovanna Juan, Raoul Jean-Luc

**Affiliations:** ^1^ Department of Medical Oncology, Paoli-Calmettes Institute, Marseille 13273, France; ^2^ Biostatistic Unit, Paoli-Calmettes Institute, Marseille 13273, France; ^3^ Department of Digestive Surgery, Paoli-Calmettes Institute, Marseille 13273, France; ^4^ Department of Pathology, Paoli-Calmettes Institute, Marseille 13273, France; ^5^ Centre de Recherche en Cancérologie de Marseille (CRCM), INSERM U1068, CNRS UMR 7258, Aix-Marseille University and Paoli-Calmettes Institute, Marseille 13273, France

**Keywords:** pancreatic adenocarcinoma, lung metastases, prognosis, overall survival, local evolution

## Abstract

The prognosis of metastatic pancreatic ductal adenocarcinoma (PDAC) is grim, with a median overall survival of under 1 year. In our clinical practice, we observed a few cases of isolated lung metastases from PDAC with unusually long outcomes. We compared these cases in a case-control study of lung-only vs. liver-only metastases from PDAC.

From our database, we found 37 cases of lung-only metastases and paired them with 37 cases of liver-only metastases by age, tumor location and treatment.

The lung-only group differed significantly from the liver-only group with respect to the following parameters: female predominance, more metachronous cases, fewer nodules per patient, and smaller increases in tumor markers. Local invasion parameters (i.e., arterial or venous involvement) were not significantly different. The outcomes were significantly different, with a median overall survival from the occurrence of metastases of 20.8 vs. 9.1 months and a median progression-free survival of 11 vs. 3.5 months.

In conclusion, this case-control study seemed to confirm that lung-only PDAC metastases have prognoses different from those of liver-only metastases. A better understanding of the mechanisms underlying these differences will help identify abnormalities associated with tumor aggressiveness.

## INTRODUCTION

Pancreatic ductal adenocarcinomas (PDAC) frequently occur and have dismal prognoses. Despite progress in systemic chemotherapy in select patients, the median overall survival is less than 1 year [1, 2]. In our Comprehensive Cancer Center, all new or progressive cases of patients with PDAC were reviewed by a weekly tumor board dedicated to liver and pancreatic tumors. In parallel we have used tumor biopsies and resected specimens to identify gene signatures of good and poor prognoses [3, 4]. Clinically, we were surprised to observe the development of slowly growing PDAC lung metastases in a few patients. In a few of these cases, only CT scan-guided biopsies performed months after minor progression confirmed these diagnoses. We collected the cases of isolated PDAC lung metastases observed in our Comprehensive Cancer Center over a 6-year period to conduct a case-control study. Each patient was paired with a “liver-only” metastatic PDAC case to confirm these observations and to explain these discrepancies.

## RESULTS

From 2007 to 2013, we treated 582 patients with PDAC; 37 of these cases (6.4 %) had “lung-only” metastases. These cases were paired with 37 cases of “liver-only” metastases. The principal clinical characteristics and the applied treatments of these 74 patients are described in Table 1. These two groups differed in several parameters: a predominance of women (73 % vs. 43 %; p < 0.01) and delayed appearances of metastases were observed in the “lung-only” group. Most “lung-only” metastases were metachronous in contrast with “liver-only” metastases (59 % vs. 11 %; p < 0.0001). For the metachronous metastases cases, the median progression-free survivals were 13 months (“lung-only” group) and 6 months (“liver-only” group). Tumor markers (CEA and CA 19-9) were less frequently identified and were found at lower rates in the “lung-only” group than in the “liver-only” group (p < 0.04). The number of metastases was more frequently above 5 in the “liver-only” group than in the “lung-only” group (67 % vs. 43 %; p < 0.05). Other parameters, including pathological parameters (differentiation), were not significantly different. In particular, the main parameters of local invasion (arterial and venous involvement) were not significantly different between the two groups.

**Table 1:** Major clinical characteristics and administered treatments for the 2 groups of “liver-only” or “lung-only” metastases from pancreatic adenocarcinomas (F: female, M: male; CEA: carcino embryonic antigen, CA 19-9: carbohydrate antigen 19-9; ULN: upper limit of the normal)

	Liver metastases N = 37	Lung metastases N = 37	p
**Age: median (range)**	71 (50 – 87)	70 (54 – 86)	NS
**Sex: F / M**	16 / 21	27 / 10	< 0.01
**Treatment (first line):**			NS
** FOLFIRINOX**	7	7	
** Gemcitabine/5FU**	30	28	
** Destruction (RFTA, Chir)**	0	2	
**Vascular involvement:**			
**Arterial**	27 %	27 %	NS
**venous**	43 %	46 %	NS
**Synchronous/metachronous**	33 / 4	15 / 22	P < 0.001
**CEA (X ULN): median (range)**	2 (1 – 1000)	1 (1 – 3)	P < 0.04
**CA 19-9 (X ULN): median (range)**	15 (1 – 10,000)	3 (1 – 100)	P < 0.04
**# nodules: 1-5 / > 5**	12 / 25	21 / 16	P < 0.05

In the “lung-only” group, 3 patients benefited from surgical resection, and 2 recurred and benefited from radiofrequency ablation. At the end of the follow-up, 3 patients were alive with non-evolutive disease 90, 35 and 20 months after recurrence; 3 developed bone metastases and 2 developed brain metastases leading to death 49, 19, 18, 76, and 72 months after recurrence, respectively; 14 did not develop metastases at any other sites; and 15 quickly developed fatal liver metastases or peritoneal carcinomatosis.

**Figure 1:**
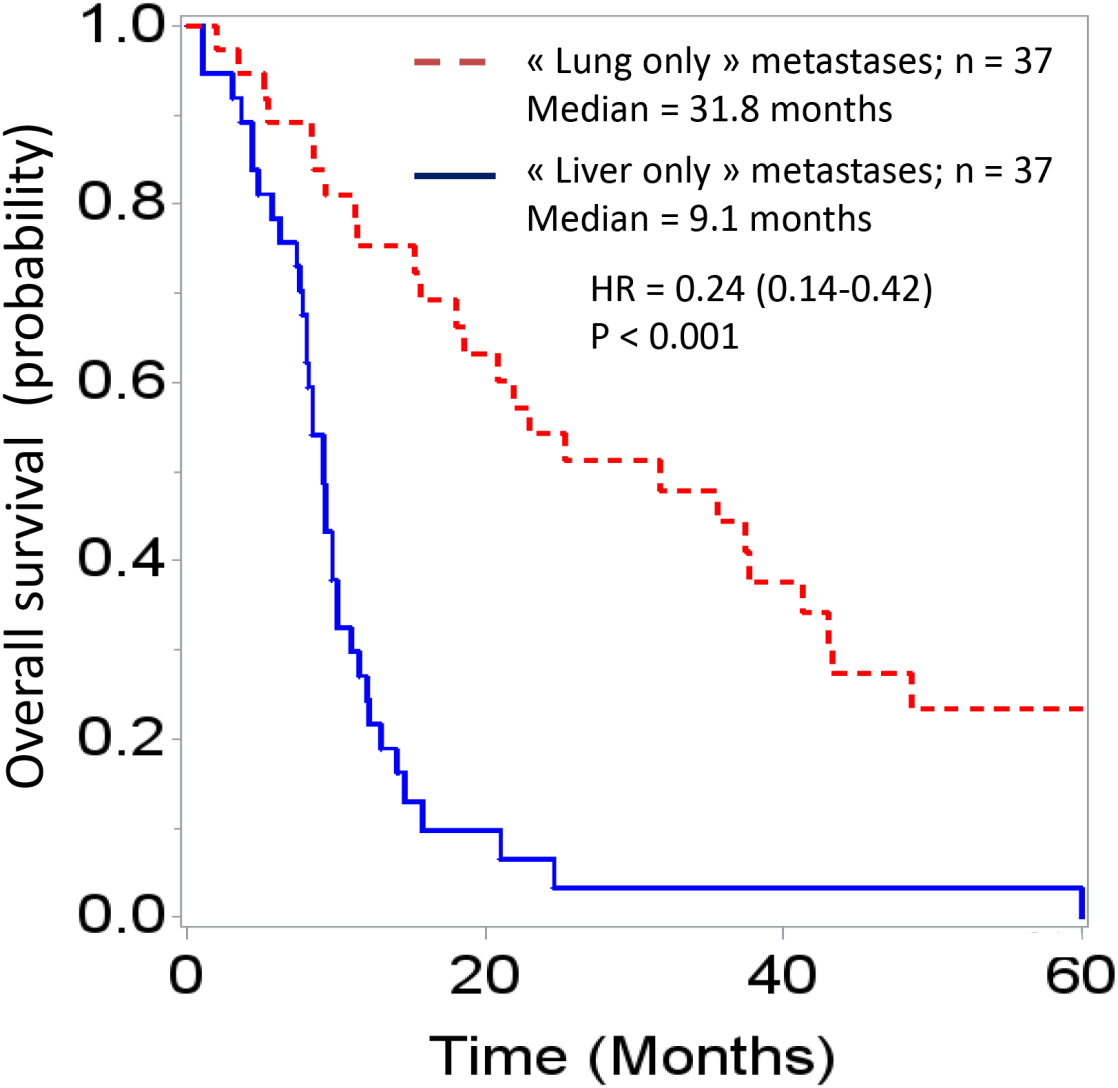
Overall survival curves of patients diagnosed with “lung-only” (n = 37) or “liver-only” (n = 37) metastases from pancreatic adenocarcinoma; t0 is the date of diagnosis of the pancreatic cancer

**Figure 2:**
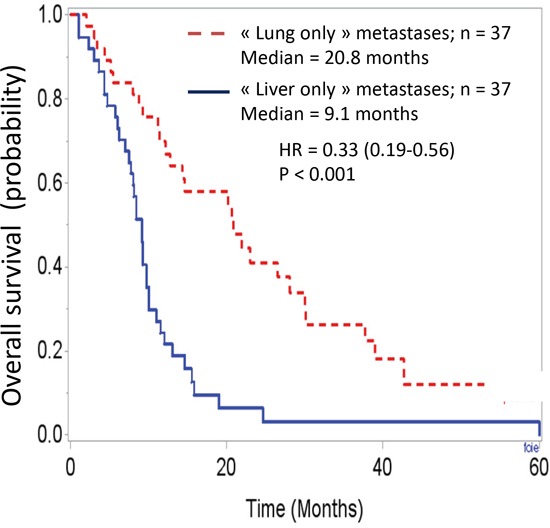
Overall survival curves in these two population, t0 being the date of diagnosis of the metastases

**Figure 3:**
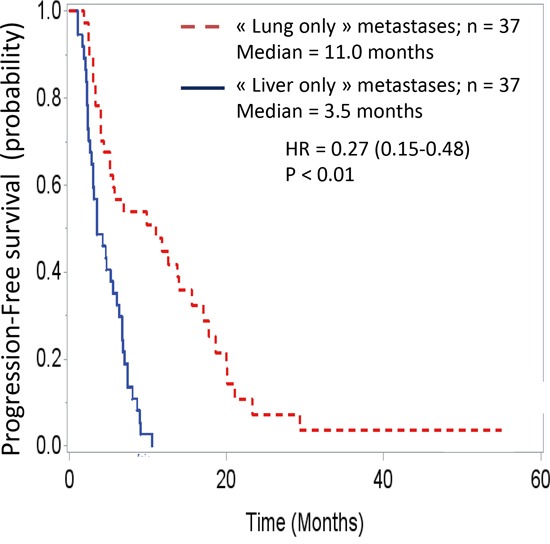
Progression free survival of these two population, t0 being the date of diagnosis of the metastases

The survival analysis demonstrated major differences between these two groups. Overall survival curves, with t0 being the date of cancer diagnosis (Figure 1), were significantly different (HR = 0.24 [0.14-0.42]; p < 0.001). The median overall survivals were 31.8 months and 9.1 months in the “lung-only” and “liver-only” groups, respectively. If t0 is set at the time of metastasis diagnosis (Figure 2), these data remain similar (HR = 0.33 [0.19-0.56], p < 0.001). The median overall survivals were 20.8 months and 9.1 months. Notably, the 2-year survivals were 41% and 6%. The survival curves regarding progression-free survival (Figure 3) were different: HR = 0.27 [0.15-0.48], p < 0.01 with median PFSs of 11 months and 3.5 months, in favor of the “lung-only” group.

## DISCUSSION

This case-control series shows that the prognosis of metastatic PDAC depended on their location. Patients with “lung-only” metastases had a 2-fold greater survival than the patients of the “liver-only” metastases group.

There were a few limitations to this analysis. First, this was a retrospective analysis; a few of the cases may have not been properly recorded in the database. Second, we did not conduct any histological studies to determine the metastatic nature of these lung nodules in all cases. However, isolated lung nodules (n = 8) benefited from resections or TDM-guided biopsies. Biopsies were performed in 7 other cases with slow growing patterns. In these cases, the pathologic descriptions were in accordance with previous histological and cytological records of the primary tumors. In the 22 other cases, the diagnoses were supported by the tumor progressions, increases in tumor markers (CEA or CA 19-9) and the absence of other malignancies.

These “lung-only” metastases were uncommon, occurring in less than 10 % of our patients. In the literature, 20 to 35% of patients in recent phase III trials [1, 2] had lung metastases. However, patients with only one metastatic site represented less than 10% of the cases [2]. The slow evolutions and long survival observed in a few of our patients prompted us to validate this observation. The simplest way to test this hypothesis was a case-control study. Therefore, we selected this method of study because a 1:1 control to case analysis seemed logical due to the major differences in outcomes. Patients with “liver-only” metastases served as controls and were paired with “lung-only” patients according to patient age, tumor location and initial treatment. These criteria were used because they were easy to access in our database and have potential prognostic value.

At the times of diagnoses, a few parameters were noticeably different between the two groups. A few of these parameters were significantly associated with “lung-only” metastases and less aggressive tumors and included metachronous lesions [1, 2], fewer nodules, and lower frequencies of tumor marker expression [5]. Paradoxically, local invasion parameters (venous or arterial involvement) were observed with the same frequency in the two groups. The patterns of PDAC evolution (local vs. metastatic) were shown to be associated with distinct genetic subtypes. In a series of 76 autopsies performed on patients who died from PDAC [6], two patterns of failure were observed: locally destructive disease and metastatic disease. Surprisingly, while 70% of the patients died from widespread metastatic disease, the cause of death was local evolution for 30% of the patients, which was associated in most cases with none or fewer than 10 metastases. Immunohistochemical analyses revealed that Dpc4 was highly correlated with the presence of widespread metastases but not with locally destructive tumors. In our study, both groups seemed to differ in the “general aggressiveness” of the disease and not in the local evolution; this accounted for the major differences in the outcomes.

The differences in the gender ratio was particularly intriguing. In the literature, PDAC occurs more frequently in males [7] (related to smoking rates). In our study, no gender-specific hormonal risk factor was correlated with the occurrence of PDAC.

Overall, this case-control study confirmed our clinical observations of major differences in outcome, overall survival and progression-free survival between the “lung-only” and the “liver-only” groups.

These differences were not attributed to any major bias in our control group. In the “liver-only” group, the median overall survival (9.1 months) and the median disease-free survival (3.5 months) were similar or were slightly higher (each control had only one site of metastatic disease, i.e., a good prognostic factor) than those observed in randomized controlled trials. The negative influences on the prognosis of liver metastases were demonstrated in 2 more recent trials [1, 2] wherein the hepatic location of the metastases were statistically identified as an independent adverse prognostic factor.

The survival observed in the “lung-only” group is impressive due to the relative similarly as that observed in a surgical series. Taking into account the survival data from the metastases diagnoses, the median OS of 20.8 months and the median PFS of 11 months were similar to the median OS and the median disease-free survival of surgery-only patients in a CONKO-001 trial (comparing patients treated with surgery-only or with surgery and adjuvant gemcitabine) [8] of 20.2 months and 6.7 months, respectively.

This slow evolution of lung metastases has recently been observed in a study of 174 patients with PDAC recurrence after surgery [9]. Of these patients, only 28 had lung metastases. These patients had a better median OS (8.5 months) than those who had recurrence in the liver (5.1 months) or in the peritoneum (2.3 months). Moreover, these lung recurrences occurred later after resection than other recurrences (8.5 months vs. 5.1 months for liver recurrences). Two cases [10] of late and unique pulmonary metastases from PDAC cured by surgery have recently been reported. In contrast, a study of resected isolated liver metastases from PDAC yielded disappointing results [11]. In this study of 15 cases, the median OS was 9.1 months and was only slightly better for metachronous cases (11.4 months).

Another single-center study of lung metastases from PDAC [12], including 31 patients who previously underwent surgery for primary tumor treatment had a few common features with our series, i.e., female predominance (65 %), late recurrences (a median time from pancreatectomy to pulmonary nodule of 29 months), and a good OS from the diagnosis of recurrence. More than 30% of the patients survived past 24 months (and even longer for 9 patients subjected to surgical resection). This case-control study succeeded in demonstrating surgery to be a curative option based on comparisons between the outcomes of resected vs. non-resected patients. The immunolabeling of Dpc4 proteins on primary tumors showed that Dpc4 loss was less frequent in patients with solitary lung nodules than in those with widespread metastatic recurrences.

According to a whole-genome sequencing analysis [13] of PDAC, an average of 119 somatic structural variants were detected per tumor. Commonly mutated genes KRAS, TP53, SMAD4 and CDKN2A were confirmed; however, many other genes from many different pathways could also be involved. This genetic heterogeneity has also been observed for metastasis-initiating cells and in phylogenic trees for organ-specific metastases [14]. In this study, the authors sequenced 3 metastases from 2 patients. A few mutations were found in all lung metastases but none were found in abdominal deposits. Another mutation was carried by all abdominal lesions but none in lung deposits. We hypothesize that the ability to overcome barriers to colonizing a given organ may depend on cancer cell subclones acquiring specific adaptive changes.

The prognostic value of site of metastases has been shown in breast cancers. For example, in a retrospective analysis of 1,038 women with metastatic breast cancer, a multivariate analysis demonstrated that age, hormonal status and metastasis site were the most relevant prognostic factors of survival. In this large study, the median overall survival of patients with bone, lung and liver metastases were, respectively, 33.2, 22.4 and 12.0 months [15]. The “seed and soil” theory first introduced in the last century remains relevant.

In conclusion, in this retrospective case-control study comparing “lung-only” to “liver-only” metastases, we observed major differences in patient profile (gender) and tumor aggressiveness (late development, less nodules per patient, and lower tumor marker expression) resulting in differences in overall survival and progression-free survival. These observations require further confirmation by other series and by conducting additional analyses to better understand pancreatic primary tumors, metastasis characteristics and outcomes. Based on these observations, we have modified our first-line options in treating patients with “lung-only” metastases with more curative options (e.g., resection and percutaneous ablation) rather than aggressive options (i.e., the use of gemcitabine over FOLFIRINOX). The effects of this approach have yet to be determined.

## MATERIALS AND METHODS

We searched our PDAC database from January 2007 to January 2013, which included 582 patients who received treatment at our institution, for cases of “lung-only” PDAC metastases. The cases were screened according to the following inclusion criteria: histological or cytological proof of malignancy of primary tumor and pulmonary metastases. The exclusion criterion was the following: demonstration of multiple sites of disease recurrence. The following patients represent the included cases.

Control patients with “liver-only” metastases were identified in the same database over the same period. These patients were paired based on age (per decade), tumor location in the pancreas (i.e., head, body or tail) and treatment (i.e., mild intensity: ablation and systemic chemotherapy based on 5FU or gemcitabine alone; or heavy intensity: oxaliplatin or irinotecan-based systemic chemotherapy). Nab-paclitaxel combined with gemcitabine was not used in our center before 2015. The inclusion and exclusion criteria were the same: the metastases have to only be located in the liver. When searching our database for the “control patients”, the first identified patient that fit the criteria was included.

Demographic and tumor parameters were expressed using median and range and were compared using the Chi-squared test. Categorical variables are shown as values or percentages. Survival curves were estimated following the Kaplan-Meier method and compared by the log-rank test.

All patient identifying information were removed before the analyses. This retrospective analysis was approved by our Institutional Review Board.
